# The Effect of a Synthetic Scent on Cheetah Behaviour

**DOI:** 10.3390/ani13030369

**Published:** 2023-01-21

**Authors:** Alexia Tommasi, Andreas G. J. Tredoux, Jacek A. Koziel, Giulia Esposito

**Affiliations:** 1 Department of Animal Sciences, Stellenbosch University, Stellenbosch 7600, South Africa; 2 Department of Chemistry, Stellenbosch University, Stellenbosch 7600, South Africa; 3 USDA-ARS Conservation and Production Research Laboratory, Bushland, TX 79012, USA; 4 Department of Veterinary Science, University of Parma, 43126 Parma, Italy

**Keywords:** *Acinonyx jubatus*, cheetah, captive breeding, volatile organic chemical, reproductive behaviour, synthetic scent, wildlife conservation, pheromones, semiochemicals, oestrus frequency

## Abstract

**Simple Summary:**

Up to date, little is known about the role of semiochemicals in cheetahs. Scientists have speculated that the concentration of VOCs potentially involved in breeding behaviour could be present at low concentrations since cheetahs’ urine emits very little odour. Therefore, the effect of a synthetic scent has been tested on female cheetahs (*Acinonyx jubatus*) behaviour with the objective of possibly increasing the frequency of reproductive (e.g., precopulatory and copulatory) behaviour displayed by female cheetahs. Furthermore, this study aimed at investigating the function of semiochemicals in reproduction in this species. The study indicates that volatile organic chemicals (VOCs) have an effect on cheetahs and their behaviour; however, additional research is required to determine the effects of specific VOCs on cheetah reproduction. “Fine tuning” of the synthetic scent could potentially aid in improving captive breeding, as well as preventing asymmetric reproductive ageing.

**Abstract:**

In cheetahs, age at first parturition correlates negatively with reproductive lifespan (asymmetric reproductive aging); therefore, breeding cheetahs at a young age is essential to maximize reproductive performance in this species. However, younger females display a significantly reduced frequency of copulatory behaviour, which negatively affects breeding. Volatile organic compounds (VOCs) are known to regulate appropriate behavioural responses in various species, including reproductive behaviour; moreover, they have proven to play a role in captive breeding methods in cheetahs, as well as mate choice. Therefore, the objective of this study was to evaluate the effect of a synthetic scent (SS) on the frequency of the five oestrous behaviour(s) (sniff, rub, roll, spray, and meow-chirp) known to be indicative of oestrus in female cheetahs. Based on the results of a previous study from our research group, five VOCs, identified in the marking fluid of male cheetahs, and known to be pheromones involved in reproductive behaviour, were used to create the SS. This was accomplished by mixing benzaldehyde, acetophenone, indole, dimethyl disulphide and phenol with (99.9%) ethanol. Seven female cheetahs were then observed for one oestrus cycle without stimulation (control) and then once again while exposed to the SS (treatment), which was sprayed on foil trays placed around the outside of each enclosure. The occurrence of the five oestrous behaviours was recorded and tallied per day of observations. Although the SS did not have a significant effect on the frequency of oestrous behaviours displayed by the females used in this study, five of the seven (71%) did show an increase in their behaviour with the SS when oestrogen concentrations were at their highest (peak oestrus), including three of the four younger females. The SS also significantly increased the sniffing behaviour in general. Although the results of this study do indicate that VOCs influence cheetahs and their behaviour, firm conclusions cannot be drawn due to the low number of animals used, as well as the significant effect the observation methods had on the results. Nonetheless, this study represents the first of this kind in cheetahs, therefore representing an important step in determining the role of VOCs in aiding breeding in captivity.

## 1. Introduction

Since the 1900s, the cheetah population decreased from over 100,000 individuals worldwide to less than 7100 [1]. Overall, the cheetah population is now registered as vulnerable with a declining trend in the population [2], which emphasizes the need for more effort in the conservation of the species [3]. In response to the swift decline in the population, captive breeding of cheetahs occurred, aiming initially at growing the population [4].

Breeding cheetahs in captivity, however, was found to be a difficult task [3,5,6,7,8,9,10]. A contributing factor to the difficulties in captive breeding is that cheetahs are prone to asymmetric reproductive ageing, which means that the length of a female’s reproductive life span correlates negatively to her age at first parturition [11]. In fact, complications with uterine integrity are the main causes of sterility in older female cheetahs, with 50% of prime-aged females (six to eight years) and above 85% of females in the older age group (nine to fifteen years) experiencing endometrial hyperplasia [12]. 

To prevent asymmetric reproductive ageing and endometrial hyperplasia in cheetahs, females need to start breeding as young adults [11], which would also improve their odds of increasing their reproductive lifespan [13]. However, despite the fact that all female cheetahs display characteristic oestrous behaviour, with five specific behaviours positively correlating with increasing oestradiol concentrations [6], it has been observed that the frequency of oestrous behaviour displayed by younger females (<4 years) and/or non-breeders, is significantly lower than older females (>6 years) and/or breeders. 

One facility that has been very effective with breeding cheetahs is the Ann van Dyk Centre in South Africa (Brits, Northwest, South Africa). The best way to detect a female cheetah in oestrus was found to be observing the female for any behavioural changes towards the males, which are allowed to roam in a walkway between the female enclosures known as lover’s lane [14,15]. With this approach, the males can sniff around to determine which female is in oestrus [15]. Furthermore, the males’ presence has been observed to initiate the oestrous cycle in females [14]. 

Another successful breeding method includes the rotational utilization of one enclosure by a male and a female cheetah. No direct physical or visual exposure is allowed at first and the animals are observed for any reaction to each other’s scent. The cheetahs are only allowed visual exposure to one another once attraction to the other’s scent is displayed, and the male has started yipping/stutter-calling. Physical introductions are then only allowed if the female does not display any hostility towards the male, however, if the female does not display oestrous behaviour after physical introductions, they are once again separated [16].

In addition to proving that cheetahs can breed well in captivity without the use of artificial reproductive techniques, the breeding results achieved from both methods also emphasize the fact that semiochemicals are involved in cheetah courtship. 

Scent marking releases a large amount of semiochemicals that create a scent mark [17]. The purpose of semiochemicals in a scent mark is to indicate territory, identify larger predators and food/prey in the vicinity, identify neighbouring animals, signal warnings, and entice the opposite sex [9,18,19,20,21]. Furthermore, data on the sex, reproductive status and age of an animal can also be attained from their scent mark [19]. Scent deposition has been observed in numerous species as a tool for communication. In mammals, this occurs mainly by secretions from scent glands on the skin, cheeks, chin, and tail, as well as urine [6,17,22].

Although numerous studies have been performed to discover the role that semiochemicals play in both domesticated and wild felids [17,18,19,23,24,25,26,27], little is known about their role in cheetahs. In fact, it has been speculated among scientists that the lack of interest towards cheetah urine is presumably due to the limited odour it releases [28], which leads to further speculation that the volatile organic compounds (VOCs) possibly related to breeding behaviour could be present at low concentrations [29]. 

A recent study proved the olfactory role of semiochemicals in mate choice by female cheetahs by using the voided urine of several males [30], thus proving the participation of semiochemicals in the urine in enticing the opposite sex. It has also been proven that the presence/odour of a male cheetah can trigger the oestrous cycle in females [14,16].

Recently, a study was conducted to detect the volatile organic profile of marking fluid collected from non-breeding and breeding male cheetahs using gas chromatography–mass spectrometry (GC-MS). It was discovered that five VOCs (benzaldehyde, acetophenone, indole, phenol and dimethyl disulfide), previously identified in other feline species, such as lions and tigers, and known to play a role in attraction/sexual behaviour in other species [17,18,20,21,27,29], were commonly identified in all the males used in the study. Unfortunately, the function of these VOCs in feline communication and behaviour is currently unknown.

Therefore, the objective of this study was to test a synthetic scent, containing the five previously mentioned VOCs, on the frequency of oestrous behaviour(s) displayed by female cheetahs.

## 2. Materials and Methods

### 2.1. Ethical Approval

The study has been approved by the Research Ethics Committee: Animal Care and Use of Stellenbosch University (project number ACU-2019-10425), Stellenbosch, South Africa.

### 2.2. Preparation of the Synthetic Scent

Dimethyl disulphide, indole, phenol, benzaldehyde and acetophenone were chosen as the components of the SS since they were previously identified in all male cheetahs [29] and are known to be pheromones involved in attraction/sexual behaviour in other species. Tommasi et al. [29] reported relative concentrations of these five volatile organic compounds (VOCs) in the marking fluid (MF) of non-breeding and breeding male cheetahs. Therefore, for the purpose of this study, the concentration of each VOC used in the synthetic scent was calculated based on the results of the breeding male’s MF. 

The relative concentrations in Tommasi et al. [29] were calculated by dividing the analyte peak area by the peak area of the internal standard (which was added to each sample at a concentration of 50 mg/L), thereby resulting in a surrogate “concentration” and would need to be converted into a unit measurement for this study. As reported by Cuadros-Rodríguez et al., [31], a roughly estimated concentration (EC, in mg/L) can be obtained through the use of a single-point calibration approach based on the internal standard. This approach was used to obtain the EC of each VOC for preparation of the synthetic scent. A calibration factor (CF) was determined as:

CF = x_std_/y_std_

where x_std_ is the concentration of the internal standard (3-octanol, in mg/L), and y_std_ is the instrumental response for the standard (expressed as the peak-area thereof); by extension to other VOC’s (assuming a linear calibration intersecting the x-axis at zero):

EC (or x_voc_) = CF (or x_std_/y_std_) × y_voc_

with y_voc_ being the instrumental (detector) response for the VOC (expressed as the peak-area) and x_voc_ being the concentration (in mg/L) of the VOC [31].

According to this, the average EC of these five VOCs from the MF of the breeding males was calculated to be as follows: benzaldehyde = ±36 mg/L, dimethyl disulphide = ±2.5 mg/L, phenol = ±2 mg/L, acetophenone = ±16 mg/L, indole = ±1.5 mg/L.

The synthetic scent was then made using, unless differently specified, chemicals obtained from Sigma-Aldrich Chemicals, South Africa. The solid/liquid form of each of the aforementioned VOCs was weighed out and mixed with 2 L of absolute ethanol (LiChrosolv^®^ Ethanol, 99.9%). The exact amount of each VOC used in the final SS can be seen in Table 1. 

Ethanol was chosen as the medium because most of the VOCs were in solid form at room temperature and, therefore, needed to dissolve in order to combine. Furthermore, ethanol evaporates quite quickly [32], thus leaving on a sprayed surface only the scent from other compounds included in the mixture rather than the scent of the ethanol. For this reason, ethanol is commonly used as a solvent in the pharmaceutical and cosmetic industries [33], especially to create fine perfumes [34]. Furthermore, a commercial scent known as Feliway (CEVA Animal Health, Pty, Ltd., Glenorie, NSW, Australia), which is used for domestic cats and sometimes wild felids such as tigers, uses ethanol as the solvent for the synthetic scent [35,36,37].

### 2.3. Sensory Analysis

A trained panel of 7 members conducted the sensory analysis. Each VOC was first mixed individually at the same concentration that was used for the SS and dropped on separate pieces of filter paper (Whatman #1). This was performed to determine what scent each individual VOC is associated with. They were then dropped together onto one filter paper to produce a combined scent of the mixed VOCs, which was then compared to the scent of three MF samples (from 2 non-breeding and 1 breeding male cheetah) collected from a previous study [38] to determine whether the scent (perceived by human panellists) of the mixture (SS) was more similar to that of the breeding or non-breeding males. While it is not possible to know what the SS “smells like” to cheetahs, this confirmation by a human nose used as a “detector” was considered to be the next best (yet imperfect) approach. Development of a synthetic formulation for odour-match-based validation with human panellists was recently shown to be useful for evaluating environmental odour downwind [39]. 

### 2.4. Preliminary Tests

A preliminary test, determining whether or not the VOCs could be detected by cheetahs at the concentrations used for the SS, was conducted with the selected five VOCs used for the SS. For the test 7 male cheetahs at Cheetah Outreach in Somerset West, Western Cape, South Africa (34°05′27.1′′ S 18°48′48.3′′ E) were involved. Their diet consisted of ±2 kg of a mix of chicken, rabbit, turkey & horse cut in pieces and supplemented with a vitamin and mineral supplement product (Predator Powder V-tech, South Africa). The seven males were exposed either to water (control) or to a single VOC (1 mL mixed with ethanol), sprayed with a sterile syringe on a wooden log already present in their enclosure and on separate foil trays presented to the animals by their handlers. Three repetitions per male, compound sprayed, and surface used (wooden log or foil tray) were completed, with an interval of at least 72 h between scents and repetition to ensure the complete disappearance of the previous scent sprayed. The foil trays were offered to the male cheetahs by their handlers once the ethanol had evaporated (±10 s after spraying the VOC). The animal’s immediate reactions to each scent were recorded. The wooden logs were used since they were already present as enrichment in the animals’ enclosures whereas the foil trays were presented to the males by their handlers since these males are used to being exposed to novel items as a requirement of the facility’s enrichment protocol. 

This test allowed us to confirm that the cheetah’s reactions towards the scent were not affected by either the foil trays or the logs. 

### 2.5. Experimental Design, Location and Animals

Eight female cheetahs, located at Feracare Wildlife Centre in Bela-Bela, Gauteng, South Africa (24°40′13.9′′ S 28°01′41.8′′ E) were enrolled in the study during July and August 2021. The animals classified, according to their age as younger (4 animals; 2–4 y) or older (4 animals; 6–8 y) were individually kept in their outdoor enclosures on either side of a walkway during the course of the observation period (30 × 15 m, surrounded by a fence 2 m high with a further half a metre of fencing hanging at a 45° toward the inside of the enclosure). 

At the time the observations were conducted, the female’s diet comprised chicken mince supplemented with dry cat food (IAMS™, PROACTIVE HEALTH™) and a vitamin and mineral supplement product (Predator powder, Old Chapel Veterinary Clinic, South Africa). All females were fed daily, except for the occasional “fasting day” once a week (as per the usual practice of the facility).

**Figure 1:**
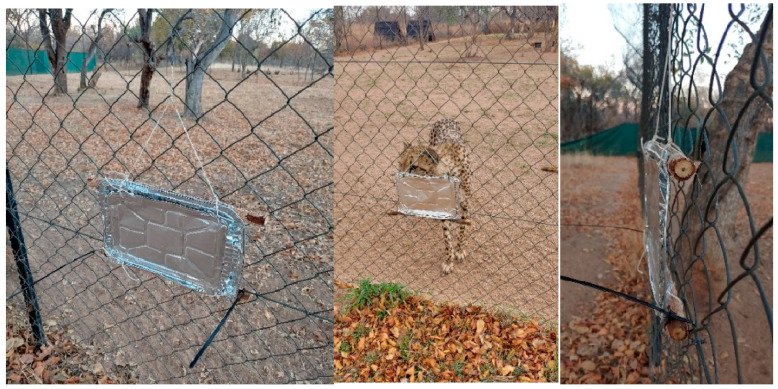
Foil tray setup.

Three foil trays with natural tree branches/sticks tied to the top and the bottom of the inside of the trays were placed around the outside of each female’s enclosure. The sticks were necessary to keep the trays away from the fence enough to allow the females to still smell the scent without being able to lick/bite the foil (Figure 1). The trays were located at nose level to ensure constant exposure to the scent while pacing around the enclosure. Cable ties were used to connect the bottom branch to the fence, and a hook connected to a string was used to keep the tray upright, while still making the inside of the tray (side facing the females) easily accessible to spray the SS. This was also completed so that the wind would not draw unnecessary attention to the trays. 

### 2.6. Oestrus Behaviour Observations

The animals were observed at least 3 times a week for 2 h between 6:30 and 9:30 am (as the sun started rising), and before feeding at approximately 10:00 am. Four of them (two for each age group) were observed in person and the other four were observed through cameras. Four cameras (S880G—3G 1080P Scouting Trail Cameras) were camouflaged in colour and were tied to the corners of the outside of the fence with the lens facing inwards. 

Females were first observed during a control period (no SS) and then again for a period of exposure to the SS (treatment – sprayed onto the foil trays). Only one day (24 h) was necessary for the females to get used to the foil trays between each phase, which is most likely due to the fact that the material of the foil trays was very similar to the aluminium bowls left in each enclosure every day.

During the SS phase, the SS was sprayed every morning before observations began at 5:30 am, and again in the evening between 16:00 and 18:00 pm. Observation periods for both the control phase and the SS phase lasted up to three weeks or until an oestrus/heat period was identified. The occurrence of five behaviours proven to correlate positively with oestradiol hormone concentrations in female cheetahs ([6], Table 2), namely rub, meow-chirp, roll, urine spray and object sniff, were recorded. The frequency at which each behaviour was displayed was then tallied per day of observation.

### 2.7. Statistical Analysis

For the preliminary test, a logistic regression was performed using the GENMOD procedure of SAS 9.4 (SAS Institute Inc., Cary, NC, USA) to assess the effect of the repetitions and the individual VOCs on the cheetahs’ behaviour, using the animals as random effect. Where applicable, repetitions and treatment (control and VOCs) with all interaction terms were included as fixed effects.

To assess the effect of the SS on the animals’ behaviour, analysis of variance (ANOVA) was conducted using the animals as random effect. Where applicable, observation method, age group and treatment (pre, post) with all interaction terms were included as fixed effects. For post hoc testing, Fisher’s least significant difference (LSD) testing was used. Normality was assessed by inspecting normal probability plots. Although in general one would expect counts not to be normally distributed, the normal probability plots were found to be acceptable. The mixed model ANOVAs were completed using the R package “lmerTest” version 3.1-0.

Every day of observations was used during statistical analysis to test the effects of the observation method (camera/in-person) on the frequency of oestrus behaviours displayed, whereas only the days included in the length of each female’s oestrus period both pre- and post-treatment (SS) were used during analysis to observe the effects of age group, SS (treatment) and the interaction between the two on the frequency of oestrus behaviours displayed.

## 3. Results and Discussion

### 3.1. Sensory Analysis of the Synthetic Scent

From the (human) sensory analysis of the five VOCs selected for the preparation of SS, benzaldehyde had a sweet, nutty scent; acetophenone had a medicinal scent; phenol smelled like tyres; dimethyl disulphide had a scent similar to rotten eggs and indole’s scent can be associated with the scent of mothballs. When comparing the scent of the five combined compounds to the MF samples, one panellist likened the scent to the non-breeding male MF and six other panellists likened the scent to the breeding male MF. This was considered to be a sufficient odour-matching from the perspective of the human sense of smell. 

The odour descriptions of benzaldehyde, dimethyl disulphide, indole, phenol and acetophenone from this study are similar to that of other studies [27,40]. The fact that six out of seven panellists (85.7%) likened the combined SS (containing the five selected VOCs) to the scent of the MF from the breeding males is unsurprising since the SS is composed of the average estimated concentration of each VOC from breeding male cheetahs [29]. The results of this analysis highlight a difference between the breeding groups in terms of the scent of the compounds when combined.

### 3.2. Immediate Reactions Observed to the Components of the Synthetic Scent

During the preliminary test with the foil, four out of the seven male cheetahs hissed after smelling the benzaldehyde, three backed away and avoided the acetophenone and one male avoided the dimethyl disulphide. No males showed any behavioural reaction towards indole and phenol. The most likely reason for this, as well as the low number of reactions towards dimethyl disulphide, could be because these three VOCs are commonly associated with livestock manure. Therefore, since the underlying biochemistry to create these VOCs is alike (with regards to digesting meat and biomass), they end up at similar endpoints to be deposited as potentially useful VOCs [20,21,41,42,43]. Similar reactions were observed with the VOCs on the log, however, there was no significant effect of any of the VOCs on the behavioural reactions of the males, and no male displayed any reaction towards the water on the log.

The immediate reactions of the female cheetahs to the SS on the foil trays were also recorded: two females rubbed their heads/necks on the fence after sniffing the foil tray, two other females sniffed a foil tray once or twice, and one female sniffed a foil tray six times and rubbed her head/neck on the fence after sniffing the tray once. 

The positive reactions observed from the female cheetahs to the combined SS suggest that the SS created for this study resembles the scent of a male cheetah since a female cheetah would naturally react positively to the scent of a male she likes [16,30], whereas the males, who are territorial [28], reacted negatively towards the SS. 

### 3.3. Behavioural Observations

One female had to be excluded from the study because of her not cycling during the study period. Furthermore, the recording of the meow-chirp (MC) behaviour had to be excluded from observations made through cameras because the sound produced from the camera’s recordings was not clear enough to distinguish the MC sound from those in the near environment (e.g., a bird’s chirp). However, of the four females observed in person, only two displayed MC behaviour. Results comparing the oestrus behaviour per age group, treatment and observation method are summarized in Table 3.

**Figure 2:**
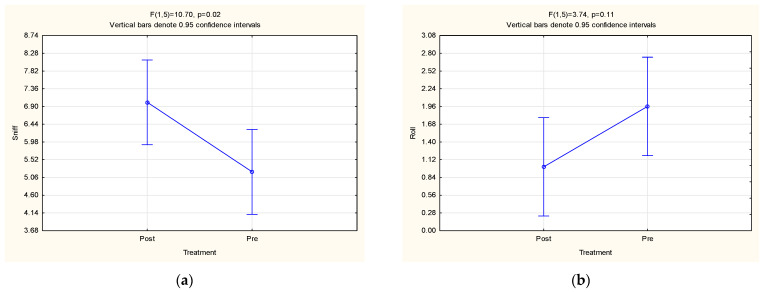
LSM ± SE results pertaining to the effect of a synthetic scent (treatment) on the (**a**) sniff and (**b**) roll behaviours of female cheetahs.

**Figure A1:**
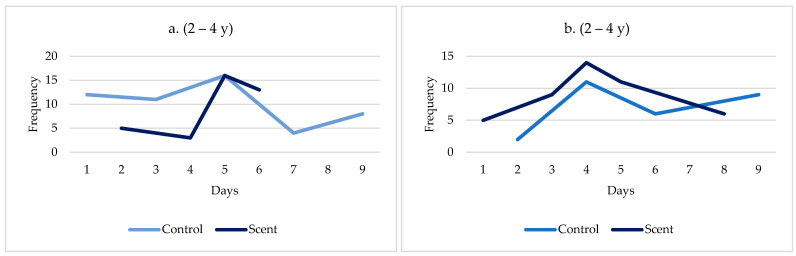

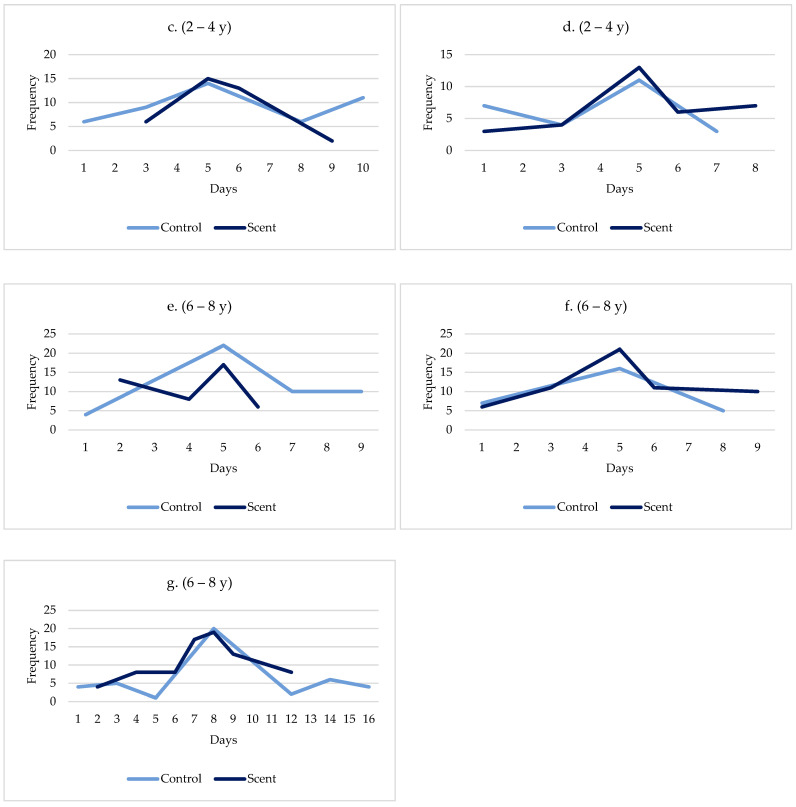
Frequency of (total) oestrous behaviours per female pre- and post-synthetic scent (excluding MC).

**Figure A2:**
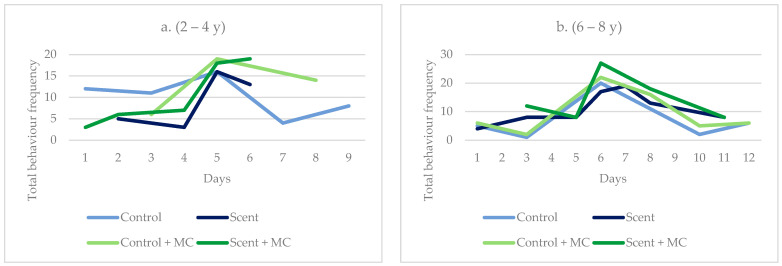
Total frequency of oestrous behaviours displayed pre- and post-synthetic scent (including MC).

The SS significantly increased the sniffing behaviour (*p* = 0.02; Figure 2a) when compared to the control period. Although the SS did not have a significant effect on the total frequency of oestrous behaviours displayed (without MC; *p* = 0.39), four out of the seven females (57.14%; Figure A1) did show an increase in their peak oestrus’ frequency with the synthetic scent. One of the older females (Figure A1g) observed in person, however, did show an increase in her peak oestrus’ frequency with the SS only when the MC behaviour was included in the recording (Figure A2b). Therefore, the total number of females that showed an increase in their peak oestrus’ frequency with the SS becomes five out of seven (71.43%). This factor emphasizes the importance of the need for extremely accurate data collection since, in this case, the MC in the inclusion of the total behavioural frequencies may influence the results. The fact that the inclusion of one female changes the percentage (of those showing an increase in their peak oestrus’ frequency) from 57.14% to 71.43% also emphasizes the need for a larger sample size.

**Figure 3:**
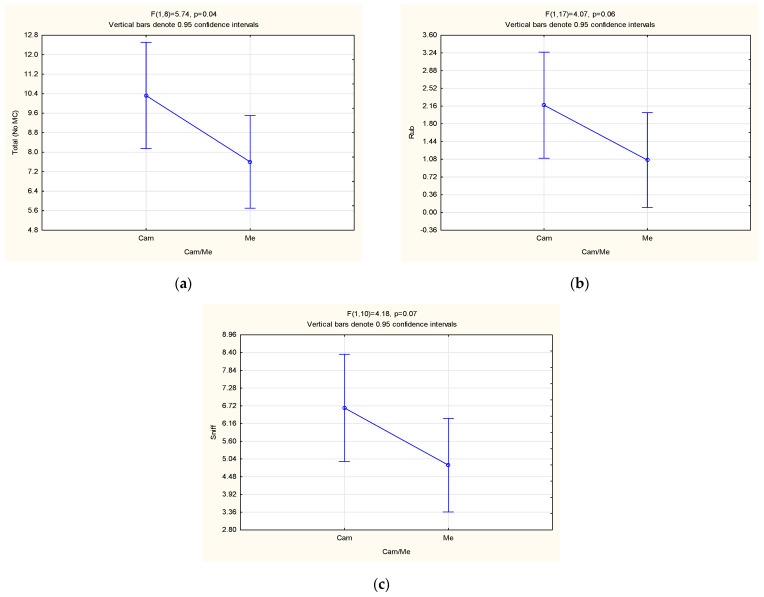
LSM ± SE results pertaining to the effect of the observation method (cam = camera and me = in person) on the (**a**) total (excluding MC), (**b**) rub and (**c**) sniff behaviours of female cheetahs.

The observation method (camera vs. in-person) had a significant effect (*p* = 0.04) on the total frequency of oestrous behaviours displayed (excluding MC). In fact, females observed through the cameras displayed a significantly higher total frequency of oestrous behaviour than those observed in person (Figure 3a). Furthermore, they tended to display a higher frequency of the rub (*p* = 0.06) and sniff (*p* = 0.07) behaviours than those observed in person (Figure 3b,c, respectively), as well. The higher behavioural frequencies of the females observed with cameras could possibly be explained by the increased accuracy of it. In fact, when the animals were observed in person, one operator was observing two cheetahs at the same time. Therefore, the operator could have missed some behaviours. With the cameras, the animals were recorded, thus increasing the accuracy of observation.

**Figure 4:**
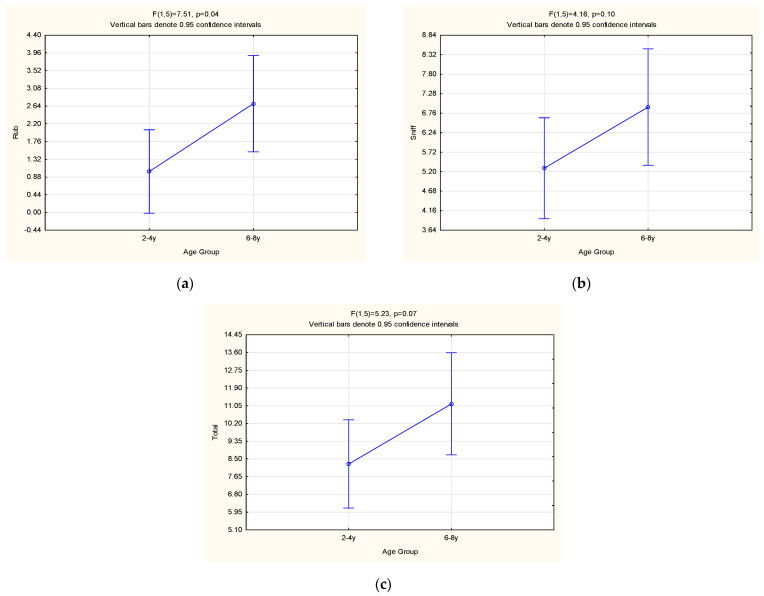
LSM ± SE results pertaining to the effect of the age group on the (**a**) rub, (**b**) sniff and (**c**) total (excluding MC) behaviours of female cheetahs.

Age had a significant effect on the rubbing behaviour (*p* = 0.04), with older females displaying a significantly higher frequency during oestrus than younger (Figure 4a). The same was observed as a tendency for the sniffing behaviour (*p* = 0.10; Figure 4b) and the total behaviours (*p* = 0.07; Figure 4c; excluding MC). These results are supported by those of Wielebnowski and Brown [6], which observed higher rates of rolling and rubbing in successful breeders (also of the older age group), along with a positive correlation of these behaviours with age [6]. In fact, they proved that the two main aspects influencing the frequency of oestrus behaviour displayed by female cheetahs are age and experience [6], meaning that an older female would be more responsive to a VOC (involved in reproduction) from the male MF than a younger female due to increased detection and memorization of the VOCs signal [44].

This study focused primarily on the long-term effects of the SS on the female’s behaviour rather than the immediate effect of the SS, which is the main difference between primer and releaser pheromones, respectively [27]. Other studies described in the literature made use of a control scent or water when testing the effects of a new SS, however, the females in this study only sniffed the foil trays directly 15 of the total 380 times they did sniff an object. Furthermore, the use of a cross-over design, along with the fact that the male cheetahs from the preliminary test did not react to all the VOCs on the foil trays or the logs, were considered aspects of a “control” in this study. The fact that the males did not react to the water on the log, yet still reacted to the VOCs on the log leads to the assumption that the females were also only affected by the SS as well. Nevertheless, the use of a control scent in the control period with the females would have further justified the results of this study. A future study could consider a larger sample size, facilitating the improved experimental design of control and treatment. 

## 4. Conclusions

The results obtained from this preliminary study showed no significant differences in the total behaviours between the groups, although an increased rate of oestrous behaviour was observed in the older females exposed to the SS compared to the younger ones, thus confirming that one or more of the five VOCs used for the preparation of the SS is most probably involved in cheetah reproduction/attraction; however, further studies focusing on younger animals are needed to improve the effectiveness of the SS in this group of animals. Unfortunately, the limitation of cases available, although a common bias for studies on endangered wildlife, together with the use of two different observation methods, do not allow us to draw firm conclusions on the efficacy of the SS in increasing the oestrous behaviour. 

It Is important, however, to bear in mind that this is the first study analysing the effect of a synthetic scent on cheetahs, therefore, despite all the limitations mentioned, the results of this study (including the positive and negative immediate reactions from the females and males to the components of the SS, respectively), shows that VOCs do have an effect on cheetah behaviour. Further testing and refining, increasing the length of the observation period to at least two oestrus cycle, increasing the sample size, and using only one method of behaviours recording, is needed to draw definite conclusions on the effect of VOCs on reproductive behaviour. Furthermore, exposing the females to the SS for longer periods before breeding, which increase detection of the signal and memorization [44], could also be a strategy to evaluate the effect of the SS on the female cheetah’s behaviour and occurrence of copulation. 

## Figures and Tables

**Table 1:** Synthetic scent expressed in mg of each VOC in 2 L of ethanol.

Volatile Organic Compound (VOC)	Total in 2 L of Ethanol (mg)
Benzaldehyde	68
Dimethyl disulphide	5.48
Phenol	3.95
Acetophenone	28.18
Indole	3.72

**Table 2:** Definitions of oestrus-related behaviours in cheetahs (adapted from Wielebnowski and Brown [6]; published with publishers permission 5472061483270).

Behaviour	Definition
Object rub	Rubs face, head, neck, or flanks on object (e.g., on fence, tree).
Roll	Rolls on back, rubbing the back on the ground while all paws are in the air, or rolls from one side to the other (each roll is then recorded as one occurrence).
Object sniff	Olfactory examination of ground (e.g., urine or faeces) or structures.
Urine spray	Urinating in standing position with tail raised against a vertical structure (frequently trees or huts). Visually the same as male urine spraying; however, females are not able to spray directionally like males.
Meow-chirp	Meow: a soft call, low-pitched, similar to domestic cat. Occasionally chirps would be emitted together with meows. Chirps were more high-pitched than meows and very short. However, these chirps were not as high-pitched, short, and loud as the chirps emitted when threatened.

**Table 3:** Least square mean ± SE of the effect of age group, treatment and observation method on cheetah’s oestrous behaviours.

Behaviour	Age Group			Treatment (SS)			Observation Method		
	2–4 Years	6–8 Years	*p*-Value	Pre	Post	*p*-Value	Camera	In Person	*p*-Value
Roll	1.73 ± 0.67	1.24 ± 1.29	0.36	1.98 ± 1.07	1.06 ± 0.66	0.11	1.52 ± 1.81	1.40 ± 1.72	0.42
Rub	1.02 ± 0.69	2.70 ± 1.41	0.04	1.50 ± 0.89	1.98 ± 1.71	0.38	1.57 ± 1.71	1.21 ± 1.93	0.06
Sniff	5.29 ± 1.41	6.92 ± 1.63	0.10	5.11 ± 1.73	6.87 ± 1.12	0.02	6.63 ± 3.00	4.81 ± 3.29	0.07
Spray	0.22 ± 0.41	0.28 ± 0.53	0.87	0.33 ± 0.58	0.15 ± 0.29	0.23	0.00 ± 0.00	0.24 ± 0.52	0.37
Total (No MC)	8.26 ± 1.56	11.14 ± 2.91	0.07	8.92 ± 3.18	10.07 ± 1.92	0.39	9.72 ± 3.89	7.67 ± 4.66	0.04

## Data Availability

All data supporting results and conclusions is presented within this paper, however, raw data will be made available upon request to the corresponding author.
